# Community pharmacists’ perspectives on E-pharmacy: an imminent threat or an opportunity in disguise?

**DOI:** 10.3389/fmed.2026.1757921

**Published:** 2026-03-03

**Authors:** Moteb Khobrani, Sultan M. Alshahrani

**Affiliations:** College of Pharmacy, King Khalid University, Abha, Saudi Arabia

**Keywords:** community pharmacy practice, E-pharmacy, medication errors, pharmacist perceptions, Saudi Arabia, workflow efficiency

## Abstract

**Background:**

E-pharmacy has transformed healthcare delivery, enhancing access and affordability, yet its impact on community pharmacists remains debatable. Although these advancements improve efficiency and reduce errors, they limit pharmacist-patient interactions, raising concerns about medication adherence and healthcare quality.

**Objective:**

This study assessed community pharmacists’ perceptions of e-pharmacy as a potential threat or opportunity. It explored the challenges, barriers, and opportunities it presents and its impact on professional roles, business sustainability, and patient care.

**Methods:**

A cross-sectional questionnaire-based study was conducted between June and September 2024 among licensed community pharmacists. A total of 305 pharmacists participated. Chi-square (χ^2^) and Spearman’s rank correlation tests examined differences in pharmacists’ perceptions of e-pharmacy using SPSS version 24, with statistical significance set at *p* < 0.05.

**Results:**

Significant associations were observed between demographic variables and pharmacists’ perceptions of e-pharmacy. A significant association was found between age and perceptions of e-prescribing challenges (χ^2^ = 10.85, *p* = 0.012, *V* = 0.19), with pharmacists over 50 years showing greater concern (75.0%). Pharmacists in chain pharmacies demonstrated more favorable attitudes (62.7%) than those in private pharmacies (59.5%) (χ^2^ = 8.24, *p* = 0.016, *V* = 0.16). Younger pharmacists (20–30 years) held more positive attitudes toward e-pharmacy than those over 50 (χ^2^ = 12.14, *p* = 0.007, *V* = 0.20).

**Conclusion:**

Community pharmacists play a vital role in mitigating e-pharmacy errors and adapting to digital workflows. Policymakers must partner with pharmacists to enhance efficiency while preserving their essential role in patient care. Although conducted in the Aseer region, these findings provide exploratory insights that may inform, rather than represent, broader national discussions on e-pharmacy regulation and integration.

## 1 Introduction

The fast-evolving e-pharmacy has significantly transformed healthcare delivery, improving access, affordability, and medication availability (1). The COVID-19 pandemic accelerated the adoption of digital pharmacy services as patients sought online options due to limited access to traditional pharmacies (2, 3). While e-pharmacy offers numerous advantages, such as quicker access to medications and cost savings, concerns persist regarding patient safety, job security for pharmacists, regulatory oversight, and the risk of counterfeit drugs (4, 5). Community pharmacists, who traditionally provided medication counseling and chronic disease management, now face a changing landscape where digital technologies and automation redefine their roles (6, 7). Recent studies in Gulf Cooperation Council (GCC) countries have similarly reported evolving professional roles and regulatory challenges associated with digital pharmacy services (8, 9).

AI-driven e-pharmacy platforms have automated key pharmacy functions like prescription verification, medication dispensing, and adherence monitoring (10). Although these advancements enhance efficiency and reduce errors, they limit pharmacist-patient interactions, raising concerns about medication adherence and overall healthcare quality (11). Independent pharmacists struggle to compete with large e-pharmacy corporations that offer lower prices, home delivery, and promotional discounts, leading to decreased foot traffic and financial uncertainty for small pharmacies (12). Many small businesses risk closure without integrating digital services (13).

The upsurge of illegitimate online pharmacies increases patient safety risks (14). Studies indicate that an estimated 95% of online pharmacies operate illegally, dispensing prescription drugs without proper identification, thereby raising the rate of spurious and counterfeit medications (15, 16). These counterfeit drugs can have improper dosages or toxic ingredients, leading to serious health consequences, such as treatment failure and drug side effects (17, 18). The absence of pharmacist control in online sales increases the risk of self-medication and toxic drug interactions (19). In addition, it is challenging to regulate cross-border e-pharmacy platforms since many illegal online pharmacies are based in jurisdictions with ineffective regulatory systems, making it harder to guarantee drug authenticity and safety (20).

E-prescribing has become integral to modern pharmacy practice, allowing physicians to transmit prescriptions electronically (4). While this reduces errors from handwritten prescriptions, issues such as system incompatibility and delayed processing still affect patient care (21, 22).

Job security concerns persist as automation and AI technologies expand (23). Tools such as robotic dispensing and computerized counseling have replaced many routine pharmacy tasks (24). Independent owners also face growing financial risks competing with major e-pharmacy providers (25).

Despite these challenges, e-pharmacy offers new professional opportunities. Telepharmacy supports remote consultations and medication management in underserved areas (26). Emerging technologies such as AI-based adherence tracking and blockchain-enabled prescription validation enhance medication safety and efficiency (10, 27). Saudi Arabia’s Vision 2030 emphasizes digital transformation in healthcare, including the regulation and expansion of e-pharmacy (28). Comparable regional research from Qatar has shown that technology acceptance, regulatory trust, and professional concerns influence e-pharmacy adoption among consumers and healthcare providers (29). However, there remains limited research exploring how Saudi community pharmacists perceive this transformation. Clarifying their perspectives is vital to guide safe integration and maintain their role in patient care (30). Community pharmacists were chosen as the focus of this study because they represent the first point of patient contact and are most affected by online dispensing models.

Informed by the Technology Acceptance Model and socio-technical systems theory, this study considers how perceived usefulness, ease of use, organizational context, and professional role expectations shape pharmacists’ responses to digital pharmacy services.

This study explored whether community pharmacists in Saudi Arabia (specifically in the Aseer region) perceive e-pharmacies primarily as a threat to their professional roles or as an opportunity for growth.

This exploratory study specifically sought to address the following research questions: (1) What are community pharmacists’ attitudes toward e-pharmacy in terms of perceived threats and opportunities? (2) What challenges and barriers do community pharmacists associate with e-pharmacy adoption? (3) What professional and service-related opportunities do pharmacists perceive in relation to e-pharmacy? (4) Are pharmacists’ perceptions associated with demographic and professional characteristics?

## 2 Methodology

### 2.1 Study design

A cross-sectional approach was used, and data were collected using a survey questionnaire. This study was designed as an exploratory investigation aimed at describing community pharmacists’ perceptions rather than testing predefined hypotheses. The study was conducted in community pharmacies in the Aseer region for 6 months. The participants in the survey self-administered the questionnaire. The study employed a simple random sampling approach to select community pharmacies for participation, ensuring each pharmacy had an equal chance of inclusion. A list of registered community pharmacies in the Aseer region was obtained, and pharmacies were selected using a computer-generated random sequence.

### 2.2 Study population

The survey questionnaire was distributed randomly to a total of 90 community pharmacies. Each community pharmacy had 2–3 pharmacists working in two shifts. Based on the most recent statistics, 747 registered community pharmacists in the Aseer region were eligible to participate and provide responses to the survey. All eligible pharmacists working in the selected pharmacies during the data collection period were invited to participate.

### 2.3 Eligibility criteria

Licensed community pharmacists currently practicing in retail or chain community pharmacies in the Aseer region were included in the study. Exclusion criteria comprised pharmacy interns, temporary staff, non-practicing pharmacists, and pharmacists working exclusively in hospital or industrial settings. Participants were required to have a valid practice license issued by the Saudi Commission for Health Specialties and at least 6 months of continuous experience in community pharmacy to ensure familiarity with e-pharmacy practices.

### 2.4 Sample size calculation

Based on the sample size calculation website,^1^ the estimated number of potential participants for the study would be 252. This calculation was based on a 95% confidence interval, a 5% margin of error, and a 50% response distribution. The total number of participants in the study was 305 community pharmacists.

### 2.5 Study tool

The questionnaire was designed to collect information aligned with the study’s aim of exploring community pharmacists’ perceptions of e-pharmacy as either an imminent threat or an opportunity in disguise. The questionnaire items were developed after reviewing previous validated instruments used in similar studies on e-pharmacy and digital pharmacy adoption (4, 31, 32), and consulting three experts in pharmacy practice and research. Questions were created and refined to ensure face and content validity, addressing attitudes, perceived challenges, and opportunities related to e-pharmacy.

The questionnaire consisted of closed-ended items designed to capture respondents’ levels of agreement with statements related to e-pharmacy adoption and practice. Examples of items included: “E-pharmacy threatens the traditional role of community pharmacists,” “E-pharmacy is a positive addition to healthcare services,” and “Digital platforms improve medication adherence through reminders and tracking.” The full questionnaire is provided as Supplementary material.

### 2.6 Data collection

The questionnaire consisted of four domains: Domain I included demographic characteristics (6 items) to capture participant profiles such as age, gender, educational background, and years of professional experience; Domain II comprised 6 questions evaluating the perceptions of community pharmacists towards e-pharmacy, including whether it is seen as a disruptive threat or a valuable opportunity for innovation. Domain III Challenges and Threats of E-Pharmacy for Community Pharmacists (8 questions); Domain IV Opportunities Provided by E-Pharmacy for Community Pharmacy Services (6 questions).

A pilot study was initially conducted with 15 community pharmacists to ensure the survey’s reliability and validity. Data were collected between June and September 2024. A self-administered questionnaire was developed, and the pilot sample was assessed and validated to ensure the quality and internal consistency of the survey. The Cronbach’s alpha coefficient was calculated as 0.79, indicating good reliability. According to established guidelines, a Cronbach’s alpha value above 0.7 is considered acceptable for internal consistency. The pilot participants represented different types of community pharmacies and provided feedback that led to minor adjustments in question wording and structure. Furthermore, input from three experts in the field was sought to refine the survey process. The results of the pilot study were not included in the final analysis.

An online self-administered questionnaire was developed using Google Forms and distributed electronically to community pharmacists in Abha and other major cities within the Aseer region. To avoid duplicate responses, each participant was restricted to a single submission through system settings, and data were monitored for consistency. All questions and concerns related to the survey were addressed. Participants were provided with standardized instructions before survey completion to minimize misunderstanding of questionnaire items.

### 2.7 Study participation

Participation in the survey was voluntary, and the pharmacists were not required to provide their identification or disclose the location of their pharmacy. To ensure confidentiality, a limited number of individuals conducted data collection and analysis.

### 2.8 Statistical analysis

The questionnaires were carefully reviewed to ensure they were complete and accurate. The collected data were processed, coded, and entered into SPSS version 24 (IBM Corp., Armonk, NY, USA). Descriptive statistics summarized participants’ demographic characteristics (e.g., gender, age, education, type of pharmacy, location, and years of experience) using frequencies and percentages. Responses to ordinal-scale items assessing attitudes, perceived challenges, and opportunities were treated as ordinal data; thus, medians and interquartile ranges (IQRs) were reported to describe central tendencies and variability. The Chi-Square Test (χ^2^) of Independence was used to examine associations between demographic variables (e.g., gender, age group, type of pharmacy) and outcomes such as positive vs. negative attitudes and high vs. low perceptions of challenges. A *p*-value < 0.05 was considered statistically significant. To compare perceptions of opportunities across multiple demographic groups (e.g., age groups, educational levels, years of experience), the Kruskal-Wallis H Test was conducted, which was appropriate for ordinal data without assuming a normal distribution. Where significant differences were found, post-hoc analyses were performed to identify specific group differences. In addition, effect sizes were calculated using Cramér’s *V* for chi-square tests and eta-squared (η^2^) for Kruskal–Wallis tests to assess practical significance.

Ordinal logistic regression analysis was conducted to identify predictors of negative attitudes and high perceptions of challenges. The dependent variables were categorized as “high” versus “low” perception scores based on median splits, while independent variables included gender, age, educational level, type of pharmacy, and years of experience. This method was selected because the outcome variables were ordinal rather than continuous, making it appropriate for modeling ordered categorical data. Statistical significance was set at *α* < 0.05.

Non-parametric tests were selected because Likert-scale responses produce ordinal data, and preliminary data screening indicated non-normal distributions. These methods are appropriate for analyzing skewed data and do not require assumptions of normality.

### 2.9 Ethical approval

This study was approved by the research ethics committee of King Khalid University (ECM# 2025–110). Participants provided their information before starting the survey, and they were informed that all data obtained from them would be kept confidential throughout all steps of the study.

## 3 Results

### 3.1 Demographic characteristics

A total of 305 community pharmacists participated in the study. Most were male (65.0%), and the most common age group was 31–40 (54.4%). The predominant educational qualification was PharmD (81.0%), with a small proportion holding a BSc Pharm (15.7%) or postgraduate degree (3.3%). Most respondents worked in chain pharmacies (78.0%), while 22.0% were employed in private pharmacies. None of the participants worked in the government sector. Most participating community pharmacists were located in urban areas (83.0%), and 72.8% had 1–5 years of experience, while only 6.2% had more than 10 years of experience. Table 1 summarizes the demographic characteristics of the respondents.

**Table 1:** Demographic characteristics of community pharmacists (*N* = 305).

Variable	Category	Frequency (*n*)	Percentage (%)
Gender	Male	198	65.0
	Female	107	35.0
Age (years)	20–30	85	27.9
	31–40	166	54.4
	41–50	36	11.8
	>50	18	5.9
Educational level	BSc Pharm	48	15.7
	PharmD	247	81.0
	Postgraduate	10	3.3
Type of pharmacy	Chain pharmacy	238	78.0
	Private pharmacy	67	22.0
	Government sector	0	0.0
Location of pharmacy	Urban	253	83.0
	Rural	52	17.0
Years of experience	1–5 years	222	72.8
	5–10 years	64	21.0
	>10 years	19	6.2

### 3.2 Attitudes of community pharmacists toward E-pharmacy

The attitudes of community pharmacists toward e-pharmacy varied. While some pharmacists acknowledged its benefits, concerns about its impact on traditional roles were evident. The median score for e-pharmacy as a positive addition to healthcare services was 3.0 (IQR: 2.0–4.0), indicating a neutral to slightly positive perception. However, the belief that e-pharmacy threatens the traditional role of pharmacists had a median score of 4.0 (IQR: 3.0–5.0), reflecting strong concern. Similarly, opinions on whether e-pharmacy aligns with ethical practices were more negative (median: 2.5, IQR: 2.0–4.0). Perceptions of improved patient access (median: 3.0, IQR: 2.0–4.0) and innovation in pharmacy practice (median: 3.0, IQR: 2.0–4.0) were moderate. The findings are presented in Table 2.

**Table 2:** Attitudes of community pharmacists toward E-pharmacy (*N* = 305).

Statement	Median (IQR)
E-pharmacy is a positive addition to healthcare services	3.0 (2.0–4.0)
E-pharmacy threatens the traditional role of community pharmacists	4.0 (3.0–5.0)
E-pharmacy improves patient access, especially in underserved areas	3.0 (2.0–4.0)
E-pharmacy aligns with ethical pharmacy practices	2.5 (2.0–4.0)
E-pharmacy fosters innovation and service expansion	3.0 (2.0–4.0)

IQR, interquartile range.

### 3.3 Perceived opportunities and challenges of E-pharmacy for community pharmacists

Despite concerns, pharmacists recognized some potential opportunities with e-pharmacy. The expansion of pharmacy services to underserved areas and the use of digital platforms for medication adherence tracking were rated at a median of 3.0 (IQR: 2.0–4.0). Pharmacists also viewed e-pharmacy as an avenue for professional development and a way to enhance their role in healthcare delivery (median: 3.0, IQR: 2.5–4.0), as shown in Table 3. On the other hand, the perceived challenges of e-pharmacy outweighed its opportunities. The lack of regulatory oversight was a significant challenge (median: 4.0, IQR: 3.0–5.0). Additionally, concerns about customer loyalty decline, price competition, and threats to personalized care all had a median of 4.0 (IQR: 3.0–5.0), demonstrating strong apprehension among pharmacists regarding the future of their profession in an e-pharmacy-dominated landscape, as shown in Table 4.

**Table 3:** Perceived opportunities of E-pharmacy for community pharmacists (*N* = 305).

Statement	Median (IQR)
E-pharmacy expands pharmacy services to underserved areas	3.0 (2.0–4.0)
Digital platforms improve medication adherence through reminders and tracking	3.0 (2.0–4.0)
E-pharmacy enhances the role of pharmacists in healthcare delivery	3.0 (2.5–4.0)
E-pharmacy offers opportunities for professional development and innovation	3.0 (2.0–4.0)

IQR, interquartile range.

**Table 4:** Perceived challenges of E-pharmacy for community pharmacists (*N* = 305).

Statement	Median (IQR)
The lack of clear regulations for e-pharmacies poses significant challenges	4.0 (3.0–5.0)
Decline in customer loyalty due to e-pharmacy services	4.0 (3.0–5.0)
Difficulty competing with lower prices offered by e-pharmacies	4.0 (3.0–5.0)
Threat to personalized care in community pharmacies	4.0 (3.0–5.0)
The financial sustainability of community pharmacies is compromised by e-pharmacy growth	4.0 (3.0–5.0)

IQR, interquartile range.

### 3.4 Association between demographic variables and attitudes, challenges, and opportunities

Chi-square analysis revealed significant associations between demographic variables and attitudes toward e-pharmacy. Female pharmacists were more likely to have a positive attitude (70.1%) than male pharmacists (58.1%) (χ^2^ = 4.32, *p* = 0.038, *V* = 0.12). Younger pharmacists (20–30 years: 65.9% positive attitude) were more open to e-pharmacy compared to older pharmacists >50 years (50.0%) (χ^2^ = 12.14, *p* = 0.007, *V* = 0.20). Pharmacists working in chain pharmacies (62.7%) had more favorable views than those in private pharmacies (59.5%) (χ^2^ = 8.24, *p* = 0.016, *V* = 0.16), as shown in Table 5.

**Table 5:** Association between demographic variables and attitudes toward E-pharmacy.

Demographic variable	Frequency (*n*)	Positive attitude (%)	Negative attitude (%)	Chi-Square (*χ*^2^)	*p*-value	Effect size (V)
Gender				4.32	0.038*	0.12
Male	198	58.1	41.9			
Female	107	70.1	29.9			
Age (years)				12.14	0.007*	0.20
20–30	85	65.9	34.1			
31–40	166	62.5	37.5			
41–50	36	57.1	42.9			
>50	18	50.0	50.0			
Type of pharmacy				8.24	0.016*	0.16
Chain pharmacy	238	62.7	37.3			
Private pharmacy	67	59.5	40.5			

*p-value < 0.05 indicates statistical significance.

### 3.5 Association between demographic variables and perceptions of E-pharmacy challenges

The analysis showed that male pharmacists perceived more challenges (68.2%) compared to females (58.9%) (χ^2^ = 3.96, *p* = 0.047, *V* = 0.11). Older pharmacists (>50 years) reported the highest perception of challenges (75.0%), while younger pharmacists (20–30 years: 57.6%) were less concerned (χ^2^ = 10.85, *p* = 0.012, *V* = 0.19), as shown in Table 6.

**Table 6:** Association between demographic variables and perceptions of E-pharmacy challenges.

Demographic variable	Frequency (*n*)	High perception of challenges (%)	Low perception of challenges (%)	Chi-square (*χ*^2^)	*p*-value	Effect size (V)
Gender				3.96	0.047*	0.11
Male	198	68.2	31.8			
Female	107	58.9	41.1			
Age (years)				10.85	0.012*	0.19
20–30	85	57.6	42.4			
31–40	166	65.4	34.6			
41–50	36	70.3	29.7			
>50	18	75.0	25.0			

^*^p-value < 0.05 indicates statistical significance.

### 3.6 Comparison of opportunity perceptions across demographic groups

A Kruskal-Wallis H test revealed significant differences in opportunity perceptions across demographic groups. Younger pharmacists (20–30 years) had higher mean ranks (178.6) for opportunity perceptions than older pharmacists (*H* = 9.35, *p* = 0.025, η^2^ = 0.021). PharmD holders (185.2) saw more opportunities than BSc holders (*H* = 8.72, *p* = 0.031, η^2^ = 0.022). Additionally, pharmacists with 1–5 years of experience (172.5) perceived more opportunities compared to those with >10 years of experience (*H* = 11.43, *p* = 0.009, η^2^ = 0.031), as presented in Table 7.

**Table 7:** Comparison of opportunity perceptions across demographic groups.

Demographic variable	Groups Compared	Frequency (*n*)	Mean rank (Opportunities perceived)	Kruskal-Wallis (H)	*p*-value	Effect Size (η^2^)
Age (years)	20–30	85	178.6	9.35	0.025^*^	0.021
	31–40	166				
	41–50	36				
	>50	18				
Educational level	BSc Pharm	48	185.2	8.72	0.031^*^	0.022
	PharmD	247				
	Postgraduate	10				
Years of experience	1–5	222	172.5	11.43	0.009^*^	0.031
	5–10	64				
	>10	19				

*p-value < 0.05 indicates statistical significance.

## 4 Discussion

The sudden rise of e-pharmacy has brought opportunities and challenges for community pharmacists, especially in the changing healthcare environment in Saudi Arabia (2). While digital technologies redefine pharmacy services, pharmacists need to balance new responsibilities with protecting their historical roles in patient care (33, 34). In this study, a noticeable proportion of pharmacists expressed practical concerns about e-pharmacy’s impact on workflow and patient interaction, reflecting broader issues tied to technological readiness. This research provides insights into how the emerging role of e-pharmacy is viewed by community pharmacists, whether as an innovative opportunity or a disruptive force that threatens their professional practice. Gaining insight into these views is key to crafting policies and strategies that reconcile technology with the need to maintain pharmacists’ critical role in medication safety and personalized patient care, particularly in relation to prescription validation, medication counseling, and error prevention in increasingly digital dispensing environments.

The main objective of this research was to investigate community pharmacists’ attitudes towards e-pharmacy and determine whether it is perceived as a potential threat to their professional status or a chance for development within Saudi Arabia’s healthcare system. The research aimed to determine the main challenges, obstacles, and opportunities of e-pharmacy from the viewpoint of practicing community pharmacists. By assessing their sentiments towards online pharmacy services, the study sought to better understand how e-pharmacy influences pharmacists’ work, business viability, and patient services and insights that could inform policy and future strategies to help integrate e-pharmacy into Saudi Arabia. The findings highlight three overarching themes shaping these perceptions: technological readiness, shifts in professional identity, and wider systemic adaptation within community pharmacy practice, which mirror concerns and opportunities reported in recent Gulf Cooperation Council (GCC) literature on digital pharmacy services (8, 9).

The findings of this study indicate that e-pharmacy adoption in Saudi Arabia is met with a combination of enthusiasm and skepticism among community pharmacists. While many recognize the potential benefits of digital transformation, concerns regarding job security, regulatory oversight, and changes to traditional pharmacy roles persist (35, 36). These findings align with previous studies, which have highlighted similar concerns among pharmacists globally (35, 36). Comparable concerns have also been documented in GCC contexts, where regulatory clarity and professional role delineation remain evolving (8). Compared to international studies where e-prescribing is more established, Saudi pharmacists are still in the early stages of adaptation, with notable disparities in technology access and digital literacy (37). Moreover, concerns about medication errors due to system incompatibilities and auto-populated fields in e-prescribing software are consistent with findings from studies conducted in Europe and North America (38). Similar system-level and regulatory gaps have been reported in GCC settings, reinforcing the need for interoperable digital infrastructure and standardized oversight (9). These contextual differences may stem from varying levels of digital infrastructure maturity and regulatory enforcement, which influence how quickly pharmacists adapt to emerging technologies.

Attitudes towards e-pharmacies varied among respondents, with younger pharmacists and those working in urban settings demonstrating greater acceptance of digital pharmacy models (32). This aligns with the study by Jarva et al. (39), indicating that digital-native professionals are more receptive to health technology integration. However, experienced pharmacists voiced concerns over the reduction of pharmacist-patient interaction and the commodification of pharmacy services, an issue also noted in studies from Canada and the UK (40). Despite these concerns, there is an acknowledgment that e-pharmacies can enhance efficiency, particularly in prescription fulfillment and adherence monitoring (41). These findings are consistent with regional evidence from Qatar, where technology acceptance factors influenced adoption while professional concerns persisted (29). Interestingly, some demographic variables that might have been expected to influence attitudes, such as gender and location, did not show significant relationships in this study, suggesting that perceptions of e-pharmacy are shaped more by professional experience and role expectations than by personal characteristics. Although several associations reached statistical significance, most demonstrated small-to-moderate effect sizes, suggesting modest practical impact despite observable differences across demographic groups.

One of the greatest challenges identified in this study is the threat of job loss due to enhanced automation in pharmacy practice. The fear that e-pharmacies may cause diminished demand for conventional pharmacy jobs has been evidenced in previous studies (31). However, it has been evidenced that e-pharmacies never displace pharmacists’ needs but instead redefine their roles as validating digital scripts and maintaining medicine safety. Still, tech disparities and incompatible systems between prescribers and pharmacy sites remain the key hindrances to the free-flowing adoption of e-prescribing (21). Pharmacists also reported higher stress levels due to more time spent rectifying prescription mistakes resulting from limitations in digital systems, a challenge also identified in research on integrating e-prescriptions (42). These findings underscore the importance of protecting pharmacists’ professional scope while reallocating responsibilities toward clinical oversight, medication safety assurance, and patient education in digital workflows. These concerns reflect broader anxieties about professional identity as automation alters traditional responsibilities, reinforcing the need for structured digital training and clearer workflow standards.

Saudi Arabia’s Vision 2030 offers a window of opportunity for organized and sustainable e-pharmacy adoption (28). With digital health transformation as one of the initiative’s core pillars, the government can standardize and regulate e-pharmacy services while preserving the professional status of pharmacists (2). Telepharmacy and AI-controlled medication management systems may extend the reach of pharmacy services beyond the conventional setting, a model already successfully adopted in specific Western healthcare systems (43). In this context, community pharmacists may serve as digital gatekeepers, ensuring appropriate medication use, counseling continuity, and patient safety within hybrid e-pharmacy models. Regulatory strategies focusing on licensing, system interoperability, and AI governance will be essential to mitigate risks while enabling innovation (8). Nonetheless, interpreting these findings requires acknowledging limitations such as the cross-sectional design and the reliance on self-reported data, which may not fully capture evolving attitudes as digital systems continue to develop. Furthermore, as this study was conducted in the Aseer region, the findings should be interpreted as exploratory and may inform, rather than represent, broader national discussions on e-pharmacy integration. However, the success of such integration will be based on proper training of pharmacists and regulatory systems that ensure patient safety while encouraging innovation.

### 4.1 Implications for policy and practice in Saudi Arabia

Saudi Arabia’s Vision 2030 emphasizes digital transformation as a core pillar of healthcare modernization, positioning e-pharmacy as a strategic component of service delivery (28). The findings of this study highlight the need for regulatory frameworks that balance technological innovation with the preservation of pharmacists’ professional roles in ensuring medication safety and quality patient care. Community pharmacists are well-positioned to function as digital gatekeepers, overseeing prescription validation, monitoring drug interactions, and maintaining counseling standards within online and hybrid pharmacy models.

To safeguard professional scope and workforce sustainability, national policies should support continuous digital training programs, competency-based certification, and structured role expansion for community pharmacists. Such initiatives may enable pharmacists to transition from primarily dispensing roles toward clinical oversight and patient-centered digital services. In addition, workforce planning strategies should account for shifting skill requirements in digitally integrated pharmacies, ensuring that automation complements rather than replaces professional expertise.

Regulatory authorities should prioritize the development of standardized licensing procedures, interoperable health information systems, and transparent governance frameworks for AI-enabled pharmacy platforms. Strengthening interoperability between prescribers, pharmacies, and regulatory databases may reduce medication errors and improve continuity of care. Clear guidelines on data security, algorithmic accountability, and clinical responsibility in AI-supported dispensing systems are also essential to maintain public trust.

Furthermore, hybrid e-pharmacy models that integrate digital platforms with in-person counseling services may represent a sustainable pathway for balancing efficiency with personalized care. Such models can enhance access to medicines while preserving critical pharmacist–patient interactions. Collectively, these policy measures may contribute to safer, more resilient, and professionally sustainable e-pharmacy systems aligned with national health transformation goals.

## 5 Strengths and limitations

This study has several strengths and limitations. One limitation is its limited generalizability, as it only included community pharmacists in Aseer, which may not represent pharmacists across all regions of Saudi Arabia, particularly areas with lower urban digital adoption. This regional focus may have influenced the findings by reflecting a population more exposed to technology-driven pharmacy services. The high proportion of respondents working in chain pharmacies (78%) may have influenced attitudes toward e-pharmacy, as chain pharmacies are typically more integrated with digital platforms and corporate technological infrastructure than independent pharmacies, potentially leading to more favorable perceptions of digital service adoption compared to smaller private pharmacies. Additionally, the reliance on self-reported data introduces potential bias, as participants’ responses may be influenced by personal knowledge and expectations regarding e-pharmacy. Social desirability bias may also have contributed to more favorable reporting of attitudes toward digital pharmacy services, particularly in the context of national digital transformation initiatives, as respondents may have felt inclined to align their views with perceived institutional or policy priorities. Moreover, the study does not assess actual patient outcomes, such as medication adherence, prescription errors, or patient satisfaction, which limits the ability to evaluate the clinical effectiveness of e-pharmacy services. The study also did not account for non-response bias or sample representativeness in terms of gender and years of experience, which are typical constraints in survey-based research, and may affect the external validity of the findings.

Despite these limitations, the study has notable strengths, including its focus on a timely and relevant topic that addresses the growing impact of e-pharmacy on community pharmacists. It is among the first quantitative studies from the Aseer region examining pharmacists’ adaptation to digital pharmacy services in the post–COVID-19 context. It employs quantitative analysis using chi-square and correlation tests, ensuring objective and data-driven insights. Furthermore, the study identifies key challenges and opportunities in digital pharmacy integration, offering valuable information for policymakers and healthcare stakeholders. Its relevance is further strengthened by its alignment with Saudi Arabia’s Vision 2030 digital health transformation efforts. By situating its findings alongside emerging GCC evidence on e-pharmacy regulation and adoption (8, 9, 29), the study contributes regionally relevant insights that may guide future regulatory and workforce planning initiatives. These findings can contribute to the development of regulations and strategies that enhance patient safety while supporting the evolving role of pharmacists in digital healthcare, particularly in the context of emerging digital service models and regulatory harmonization efforts across GCC countries.

## 6 Recommendations

Some recommendations have been put forward to make e-pharmacies work successfully in the healthcare system of Saudi Arabia. It is recommended that thorough training programs be established to improve pharmacists’ digital skills and familiarity with e-prescribing systems. Such programs should be incorporated into pharmacy education curricula and continuing professional development programs. It is further recommended that policymakers enforce standardized regulations to ensure the efficacy and safety of e-pharmacy platforms. Regulatory attention must be dedicated to reducing medication errors, stopping the sale of counterfeit medications, and assuring that e-prescribing supports clinical best practices. In addition, adopting a hybrid model of integrating classical pharmacy services and e-health innovations is recommended, as this would allow pharmacists to have the necessary roles of patient counseling and drug safety while using technology to improve efficiency and access. Future research should explore the long-term impact of e-pharmacy on pharmacists’ professional roles, workflow efficiency, and service delivery through longitudinal study designs that capture changes over time. Further studies incorporating patient-reported outcomes, such as satisfaction, medication adherence, and perceived quality of care, would provide a more comprehensive assessment of e-pharmacy effectiveness from the patient perspective. Comparative studies across the Gulf Cooperation Council (GCC) or Eastern Mediterranean Region (EMR) countries are also recommended to examine contextual similarities and differences in regulatory frameworks and professional adaptation. In addition, mixed-methods research combining quantitative surveys with qualitative interviews may offer deeper insights into pharmacists’ experiences, perceived risks, and coping strategies in digitally evolving pharmacy practice. Positioning these recommendations within the broader global movement toward pharmacy digitization may also support Saudi Arabia’s potential leadership in regional regulatory innovation.

## 7 Conclusion

E-pharmacies bring with them potential benefits and profound challenges in the developing healthcare sector of Saudi Arabia. The advantages of e-pharmacies, such as safer medications, increased patient access to medication, and enhanced efficiency, render them an appealing element in healthcare modernization. With the vision of shifting towards a digitally empowered healthcare sector under Vision 2030 in Saudi Arabia, e-pharmacies can be a key player in minimizing drug errors and streamlining pharmaceutical operations. However, the changing process is being taken with alarm by pharmacists who are worried about losing jobs through automation and the erosion of pharmacist-patient contact. Technology can replace manual tasks and enable pharmacists to provide patient care through telepharmacy, medication therapy management, and clinical decision support. The study’s findings also highlight emerging patterns such as variations in acceptance across age groups and recurring workflow inefficiencies that add new insight to the understanding of e-pharmacy readiness in the Saudi context. To maximize benefits and mitigate risks, policies should focus on training pharmacists, ensuring system interoperability, and implementing regulations. A balanced approach combining technological advancements with human elements of pharmacy practice is necessary for successful integration. As this study was limited to community pharmacists in the Aseer region, the findings should be interpreted as exploratory and may inform, rather than represent, broader national perspectives on e-pharmacy implementation in Saudi Arabia. Overall, this study provides one of the early empirical assessments of community pharmacists’ preparedness for digital pharmacy integration in Saudi Arabia, offering key directions for future nationwide investigations and comparative international research.

## Data Availability

The original contributions presented in the study are included in the article/Supplementary material, further inquiries can be directed to the corresponding author.

## Footnotes

1 http://www.raosoft.com/samplesize.html, accessed on April 28, 2024.
