# Supplementary material for: Community pharmacists’ perspectives on E-pharmacy: an imminent threat or an opportunity in disguise?
Source: Front Med (Lausanne). 2026 Mar 3;13:1757921. doi: 10.3389/fmed.2026.1757921 (PMC12992040; doi:10.3389/fmed.2026.1757921)
Supplement: Supplementary file 1 [file Data_Sheet_1.pdf]

Community Pharmacists' Perspectives on E-Pharmacy: An Imminent Threat or an Opportunity in Disguise?

We are a group of researchers in College of Pharmacy, King Khalid University, conducting a research project entitled " Community Pharmacists' Perspectives on E-Pharmacy: An Imminent Threat or an Opportunity in Disguise?". The purpose of this project is to to assess This study aims to explore community pharmacists' perceptions of e-pharmacy as a potential threat or opportunity. It investigates the challenges, barriers, and opportunities posed by e-pharmacy and its impact on professional roles, business sustainability, and patient care. The findings will guide strategies to balance innovation with the preservation of community pharmacy services. Your participation in this research project is voluntary. You may choose not to participate. If you decide to participate in this research survey, you may withdraw at any time. The procedure involves filling a survey that will take approximately 5 minutes. Your responses will be confidential, and we do not collect identifying information such as your name, email address or IP address.

Study Tool:

Part 1:

Demographic and Socioeconomic Domain:

1- Gender:

A. Male
B. Female

2- Age

A. 20 to 30
B. 31 to 40
C. 41 to 50
D. Older than 50

3- Educational level *

A. Bsc. Pharm
B. PharmD
C. Postgraduate level

4- Type of Pharmacy *

A. Chain pharmacy
B. Private pharmacy
C. Government sector

5- Location of the pharmacy: *

A. Rural
B. Urban

6- Years of Experience

a- 1-5 yrears
b- 5-10 years

c- > 10 years

Part 2:

Attitudes of Community Pharmacists Towards E-Pharmacy

1. E-pharmacy services are a positive addition to the healthcare system.
2. E-pharmacy can improve patients' access to medications, especially in underserved areas.
3. E-pharmacies provide a level of convenience that community pharmacies cannot match.
4. E-pharmacies align with the principles of ethical pharmacy practice.
5. The growing popularity of e-pharmacy threatens the traditional role of community pharmacists.

Challenges and threats of E-Pharmacy for Community Pharmacists

1. The lack of clear regulations for e-pharmacies poses significant challenges to my ability to compete effectively.
2. The rise of e-pharmacies has led to a decline in customer loyalty to community pharmacies.
3. It is difficult to compete with the lower prices and convenience offered by e-pharmacies.
4. E-pharmacies threaten the traditional role of community pharmacists in providing personalized care and consultations.
5. The growing popularity of e-pharmacies makes it challenging to sustain the financial viability of community pharmacies.
6. Managing counterfeit or substandard medicines sold by unregulated e-pharmacies is a significant issue.
7. The anonymity of e-pharmacy transactions limits my ability to ensure the safe and proper use of medications.
8. The lack of technological resources and training prevents community pharmacies from competing with e-pharmacy platforms effectively.

Opportunities Provided by E-Pharmacy for Community Pharmacy Services

1. E-pharmacy systems provide opportunities to enhance medication tracking and safety.
2. Partnering with e-pharmacies can help community pharmacies expand their services (e.g., delivery).
3. E-pharmacy platforms can improve communication between pharmacists and patients.
4. Community pharmacists can use e-pharmacy data to improve medication adherence for patients.

E-pharmacies
